# Supplementary material for: Atomically dispersed iridium catalysts on silicon photoanode for efficient photoelectrochemical water splitting
Source: Nat Commun. 2023 Feb 4;14:609. doi: 10.1038/s41467-023-36335-0 (PMC9899270; doi:10.1038/s41467-023-36335-0)
Supplement: Supplementary file 1 — Supplementary Information [file 41467_2023_36335_MOESM1_ESM.pdf]

Supplementary Information

Atomically dispersed iridium catalysts on silicon photoanode for efficient photoelectrochemical water splitting

Sang Eon Jun[1], Youn-Hye Kim[2], Jaehyun Kim[1], Woo Seok Cheon[1], Sungkyun Choi[1], Jinwook Yang[1], Hoonkee Park[1], Hyungsoo Lee[3], Sun Hwa Park[4], Ki Chang Kwon[4], Jooho Moon*[3], Soo-Hyun Kim*[5], Ho Won Jang*[1, 6]

[1] Department of Materials Science and Engineering, Research Institute of Advanced Materials, Seoul National University, Seoul 08826, Republic of Korea

[2] School of Materials Science and Engineering, Yeungnam University, Gyeongsan, Gyeongbuk 38541, Republic of Korea

[3] Department of Materials Science and Engineering, Yonsei University, Seoul 03722, Republic of Korea

[4] Interdisciplinary Materials Measurement Institute, Korea Research Institute of Standards and Science, Daejeon 34113, Republic of Korea

[5] Graduate School of Semiconductor Materials and Devices Engineering, Ulsan National Institute of Science and Technology, 50 UNIST-gil, Ulju-gun, Ulsan, 44919, Republic of Korea

[6] Advanced Institute of Convergence Technology, Seoul National University, Suwon 16229, Republic of Korea

E-mail: hwjang@snu.ac.kr, soohyunsq@unist.ac.kr, jmoon@yonsei.ac.kr

CONTENTS

Supplementary Figures

Figure S1: The energy band diagrams of Ni/n-Si and Ni/ZrO_2/n-Si before equilibrium and in equilibrium under steady-state illumination.

Figure S2: HAADF-STEM image of Ir SAs/NiO thin-film catalyst observed at three different point.

Figure S3: Top-view SEM images of Ir NCs/NiO/Ni/ZrO_2/n-Si (ALD-25 cycles), Ir film/NiO/Ni/ZrO_2/n-Si (ALD-100 cycles), and Ir film-T(thick)/NiO/Ni/ZrO_2/n-Si (ALD-200 cycles).

Figure S4: XRD patterns of samples.

Figure S5: Transmittance versus wavelength spectra of samples.

Figure S6: XPS Wide scans of Ir SAs/NiO/Ni/ZrO_2/n-Si, Ir NCs/NiO/Ni/ZrO_2/n-Si, and Ir film/NiO/Ni/ZrO_2/n-Si.

Figure S7: The magnified and simplified Ir 4*f* spectra of NiO/Ni/ZrO_2/n-Si photoanodes deposited with Ir SAs, NCs, and film.

Figure S8: O 1*s* spectra of Ni/ZrO_2/n-Si and Ir SAs/NiO/Ni/ZrO_2/n-Si.

Figure S9: Mott-Schottky plots for Ni/n-Si and Ni/ZrO_2/n-Si.

Figure S10: LSV curves of Ni/ZrO_2/n-Si photoanodes with different thickness of ZrO_2 layer.

Figure S11: LSVs of NiO/Ni/ZrO_2/n-Si, Ir ALD-1cyc./ZrO_2/n-Si, and Ir SAs/NiO/Ni/n-Si.

Figure S12: The stability test of NiFe/n-Si and Ir(ALD-1cyc.)/NiFe/n-Si photoanodes.

Figure S13: LSV curves of the photoanodes with Ir SAs and Ir catalyst synthesized via 2-cycle ALD process.

Figure S14: Comparison of the onset potential, current density at 1.23 V vs. RHE, and saturation current density of Ni/n-Si, Ni/ZrO_2/n-Si, and Ir SAs/NiO/Ni/ZrO_2/n-Si.

Figure S15: Electrochemical (EC) characterizations of the fabricated anodes with IR-correction.

Figure S16: LSV curves of Ir SAs/NiO/Ni/ZrO_2/n-Si photoanode and Ir SAs/NiO/Ni/ZrO_2/p^{++}-Si anode for determining the photovoltage of the device.

Figure S17: Incident-photon-to-current conversion efficiency of the photoanodes without Ir SAs, NiO/Ni, and ZrO_2 layer.

Figure S18: Ir 4f spectra of Ir SAs/NiO/Ni/ZrO_2/n-Si photoanode before performance degradation at 120 h.

Figure S19: LSV curves of Ir SAs, NCs, and film deposited on NiO/Ni/ZrO_2/n-Si photoanodes in acidic condition.

Figure S20: Energy band diagram and equivalent circuit model for IMPS.

Figure S21: IMPS Nyquist plots at 1.0 and 1.4 V_{RHE}.

Figure S22: k_{rec} vs. potential vs. RHE (V).

Figure S23: Free energy diagrams of OER at 0 V_{RHE}.

Figure S24: Reaction energy profile of Ir SAs/NiO (100) for *OOH formation calculated with the most favorable incident OH angle.

Figure S25: Reaction energy profiles of Ir SAs/NiO (100) for *OOH formation calculated with different incident OH angles.

Supplementary Tables

Table S1: Numerical values of onset potential, saturation current density, and current density at 1.23 V_{RHE}.

Table S2: ICP-MS data

Table S3: Summary of PEC-OER performance for recently reported Ir-based PEC catalysts applied to Si photoanodes.

Table S4: Summary of PEC-OER performance for recently reported transition metal-based PEC catalysts applied to Si photoanodes.

Table S5: Numerical values of IMPS parameters at an applied potential of 1.0, 1.23, and 1.4 V_{RHE}.

Table S6: Exact values of fitted charge transfer resistance.

Supplementary Notes

Supplementary Note 1: Calculation of k_{trans} and k_{rec}.

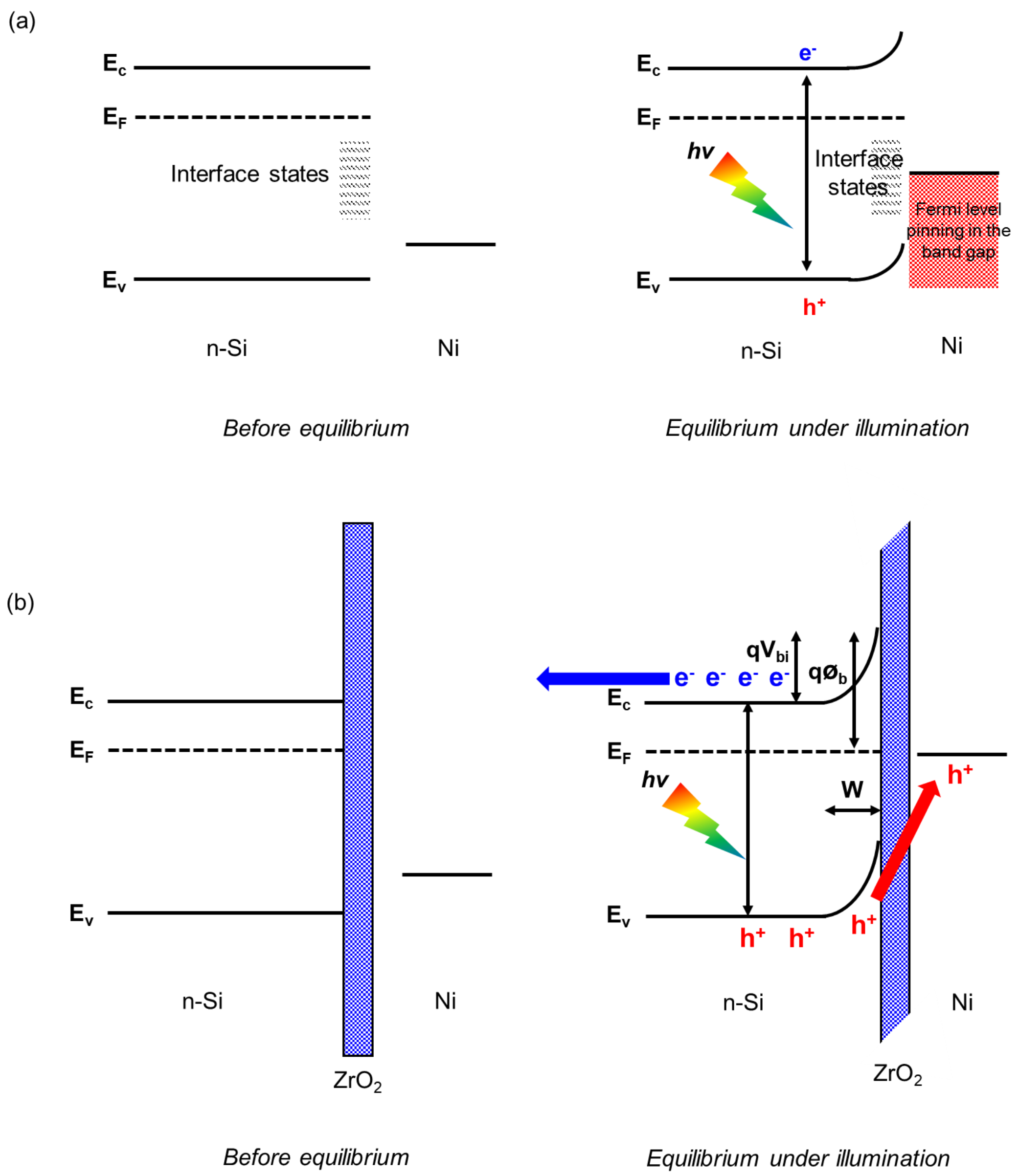

Figure S1. The energy band diagrams of **a** Ni/n-Si and **b** Ni/ZrO_2/n-Si before equilibrium and in equilibrium under steady-state illumination.

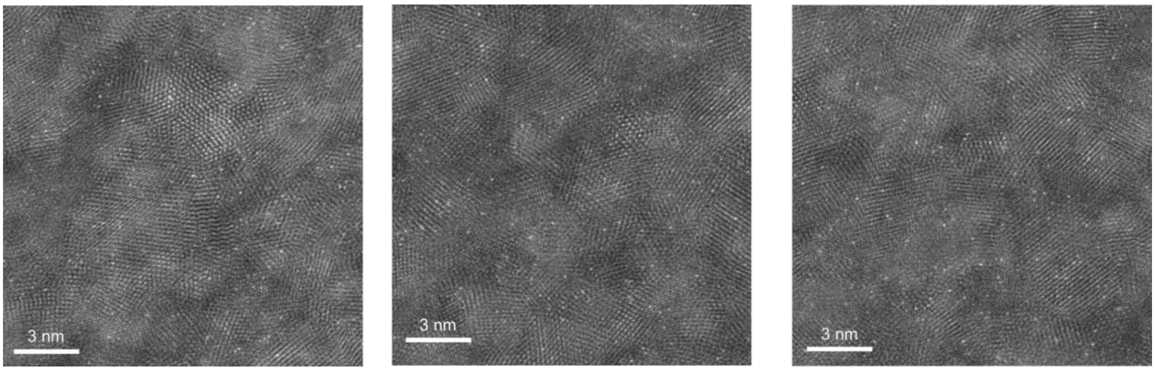

Figure S2. HAADF-STEM images of Ir SAs/NiO thin-film catalyst observed at three different point.

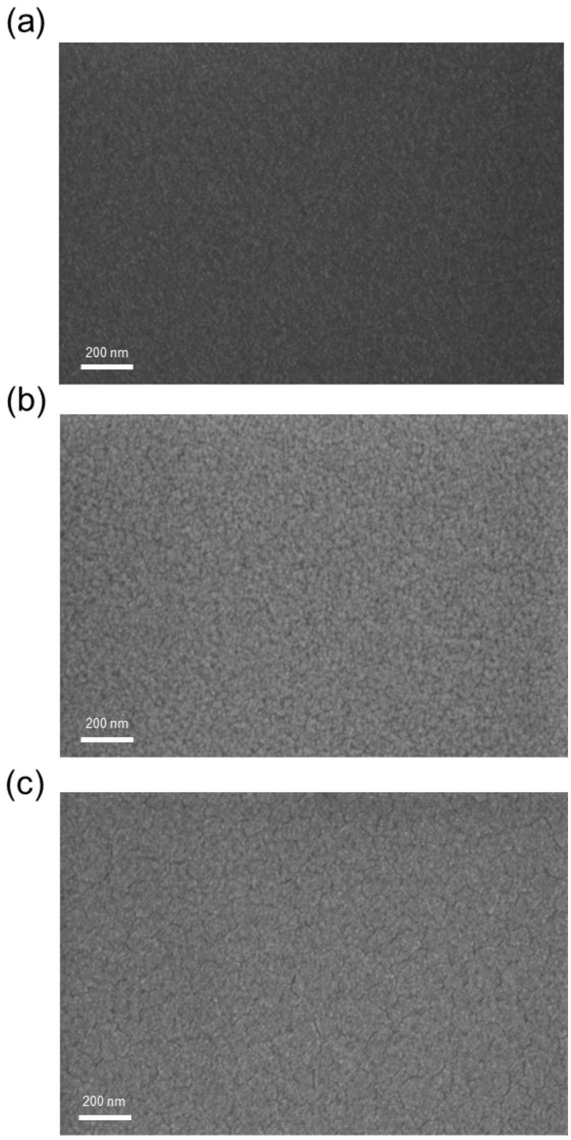

Figure S3. Top-view SEM images of **a** Ir NCs/NiO/Ni/ZrO_2/n-Si (ALD-25 cycles), **b** Ir film/NiO/Ni/ZrO_2/n-Si (ALD-100 cycles), and **c** Ir film-T(thick)/NiO/Ni/ZrO_2/n-Si (ALD-200 cycles).

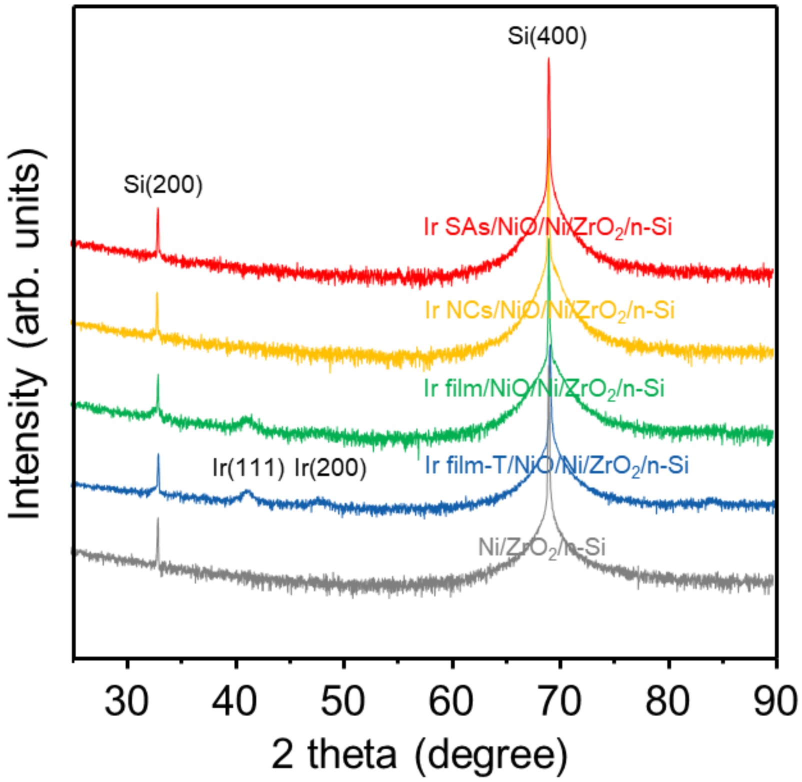

Figure S4. XRD patterns of samples.

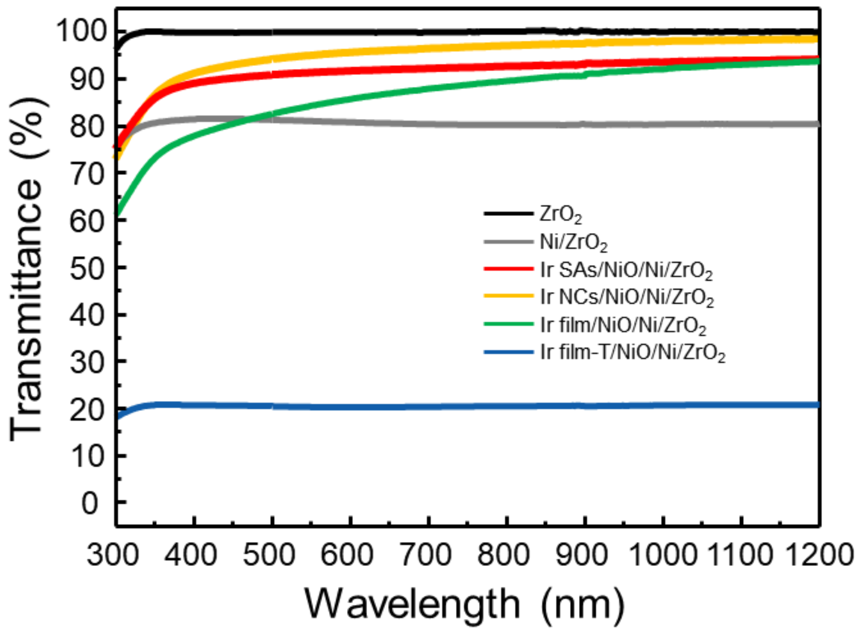

Figure S5. Transmittance versus wavelength spectra of samples.

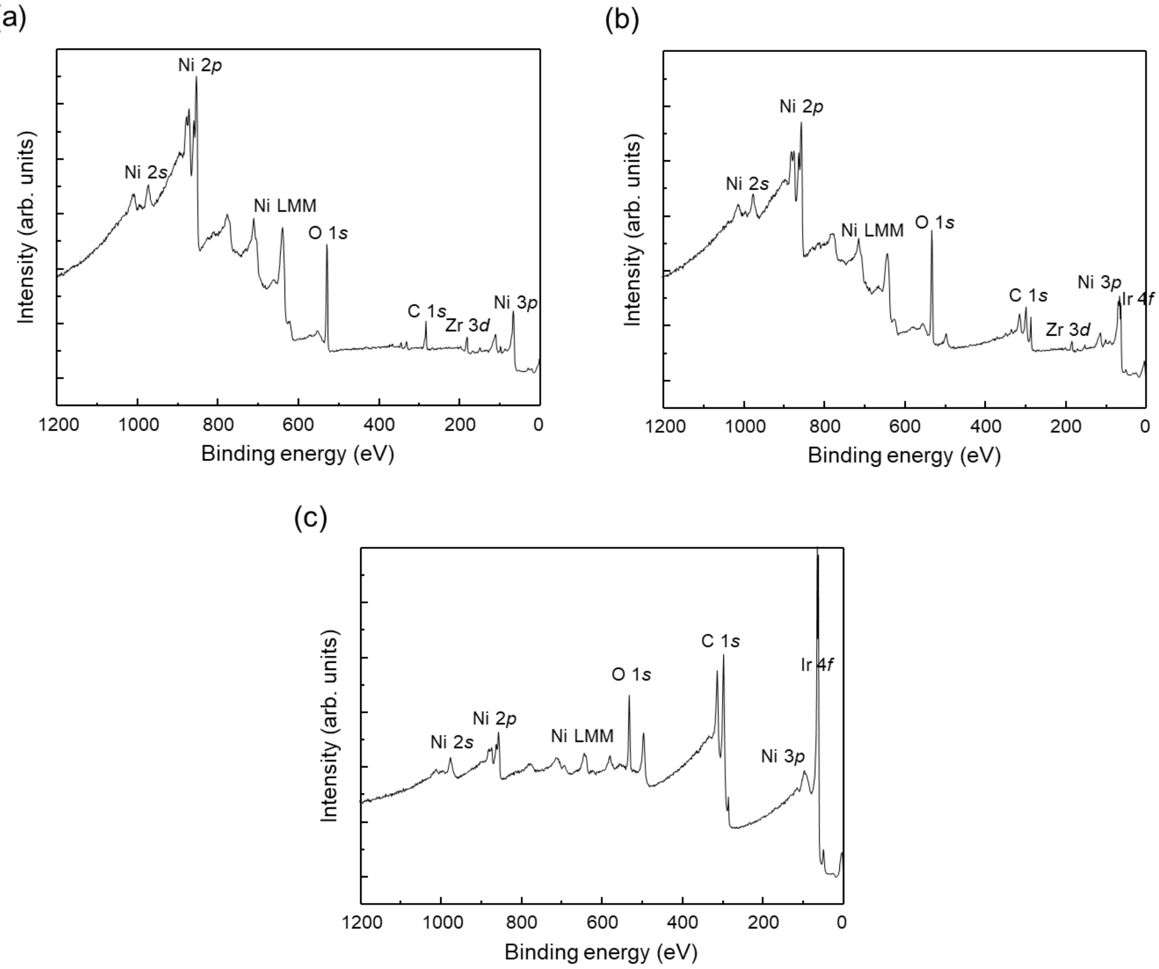

Figure S6. XPS Wide scans of **a** Ir SAs/NiO/Ni/ZrO_2/n-Si, **b** Ir NCs/NiO/Ni/ZrO_2/n-Si, and **c** Ir film/NiO/Ni/ZrO_2/n-Si.

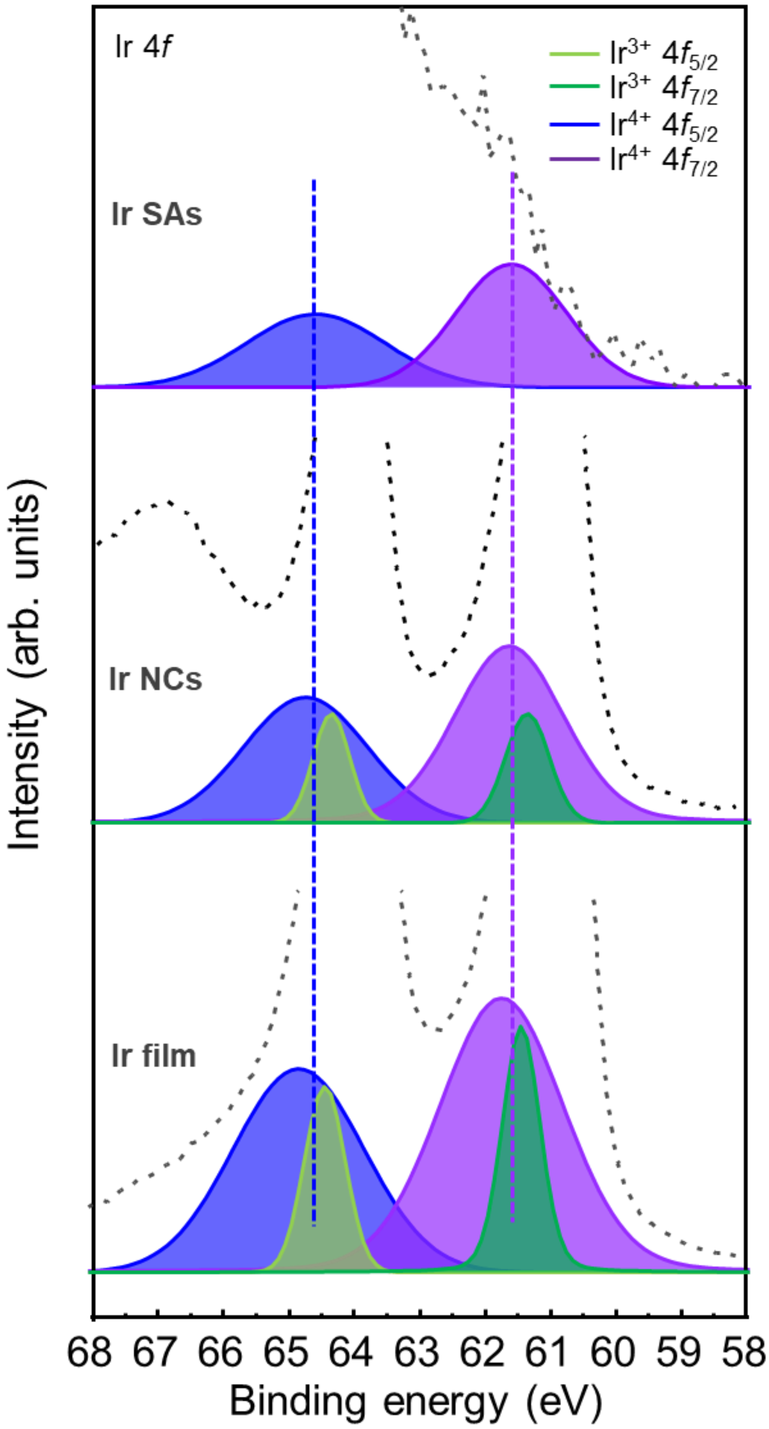

Figure S7. The magnified and simplified Ir 4*f* spectra of $NiO/Ni/ZrO_2$/n-Si photoanodes with Ir SAs, NCs, and film.

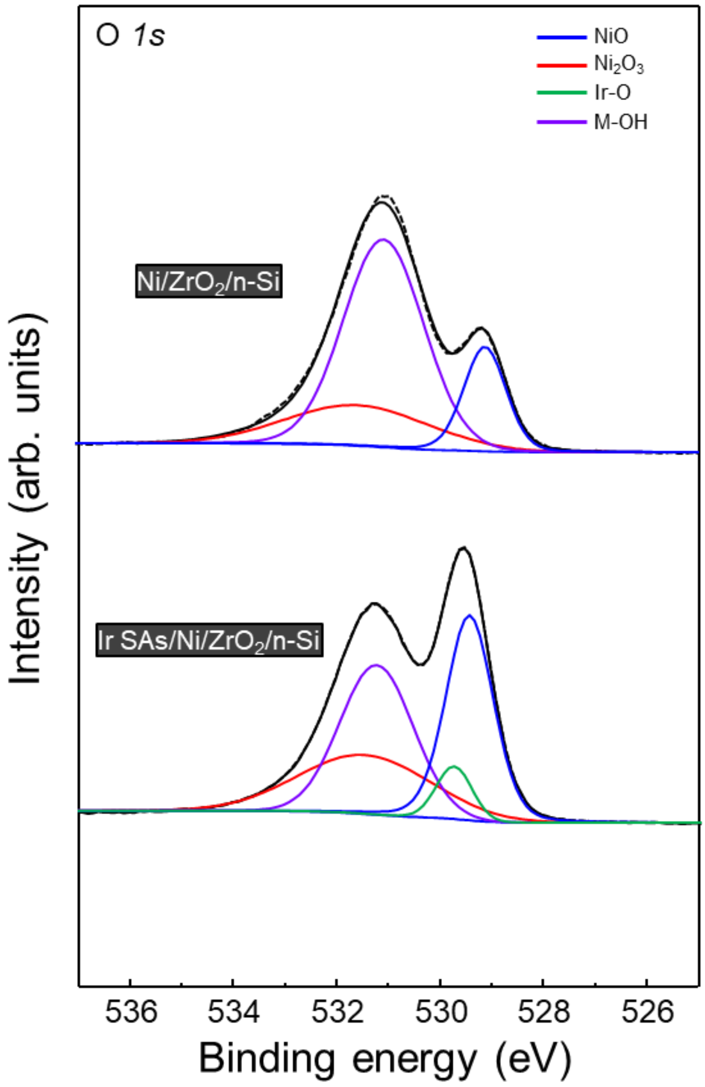

Figure S8. O 1*s* spectra of Ni/ZrO_2/n-Si and Ir SAs/NiO/Ni/ZrO_2/n-Si.

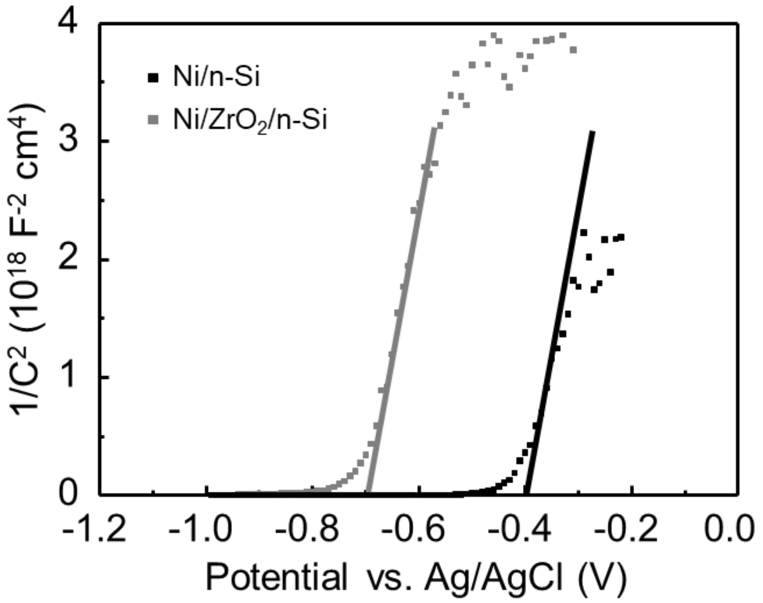

Figure S9. Mott-Schottky plots for Ni/n-Si and Ni/ZrO_2/n-Si.

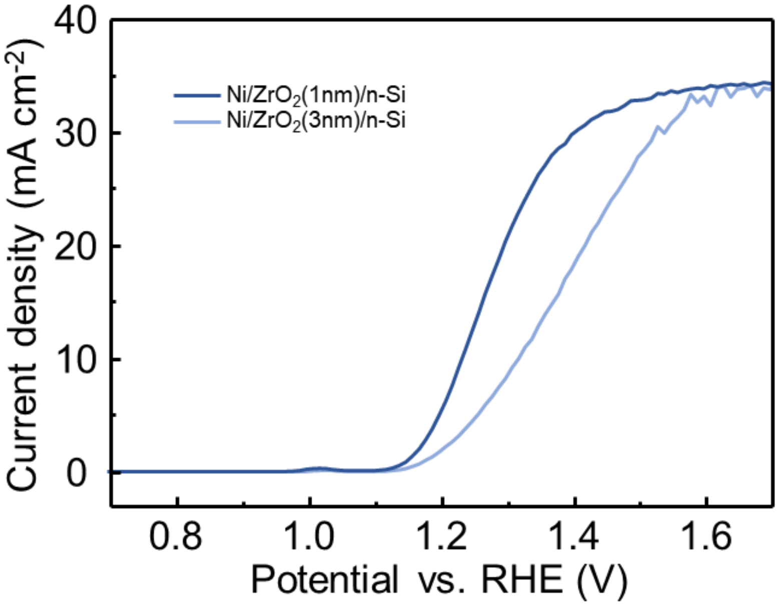

Figure S10. LSV curves of Ni/ZrO_2/n-Si photoanodes with different thickness of ZrO_2 layer.

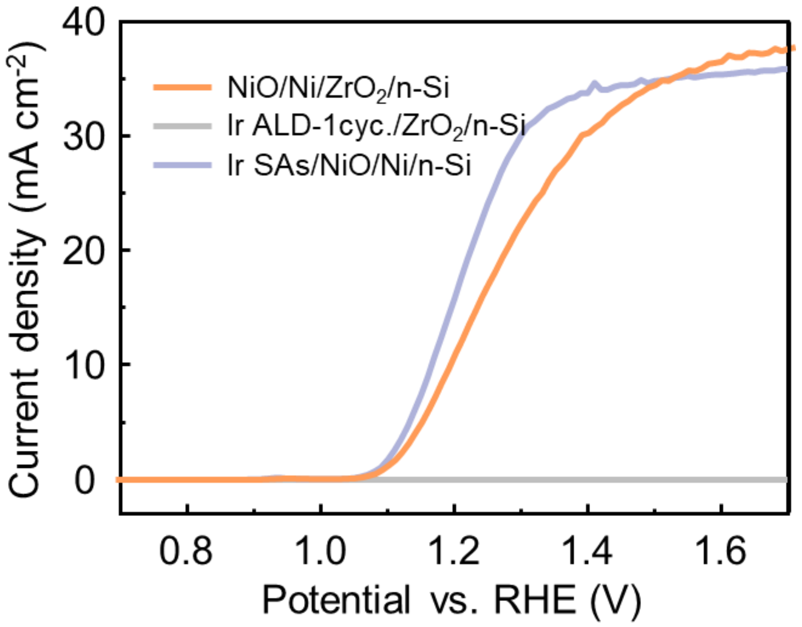

Figure S11. LSVs of $NiO/Ni/ZrO_2$/n-Si, Ir ALD-1cyc./ZrO_2/n-Si, and Ir SAs/NiO/Ni/n-Si.

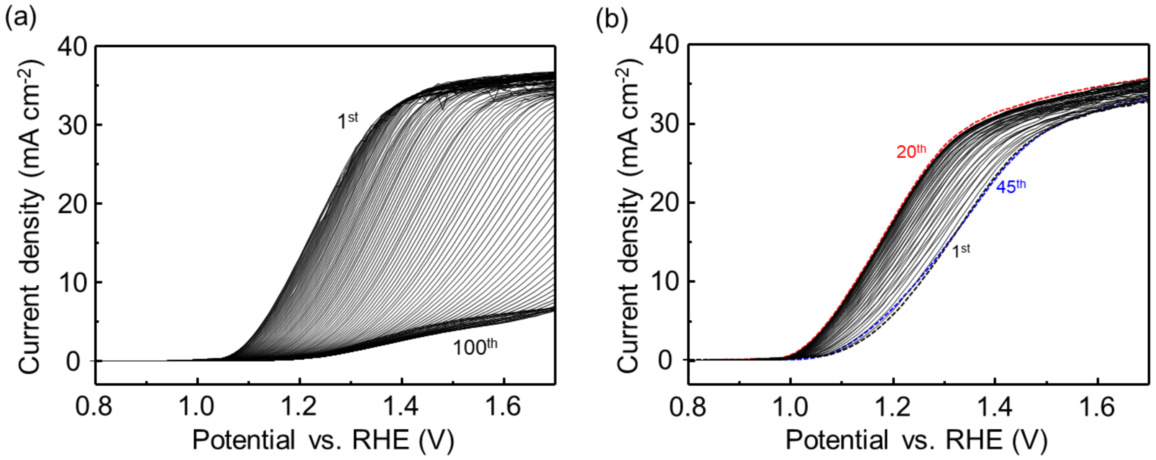

Figure S12. The stability test of **a** NiFe/n-Si and **b** Ir(ALD-1cyc.)/NiFe/n-Si photoanodes. Polarization curves of NiFe/n-Si photoanode continuously shifts positively as the CV operation increases though the remarkable performance is shown at 1st cycle. Polarization curves of Ir(1cycle)/NiFe/n-Si shifts positively after activation. The poor stability is derived from the leaching of Fe in NiFe catalysts.

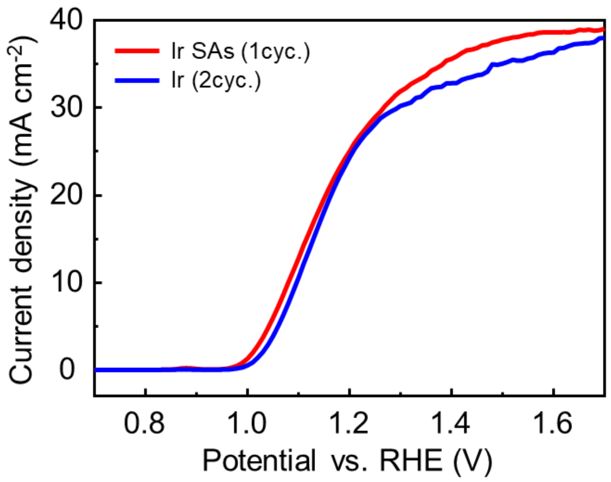

Figure S13. LSV curves of the photoanodes with Ir SAs and Ir catalyst synthesized via 2-cycle ALD process.

The catalytic activity of Ir (2cyc.) was not higher than that of Ir SAs (1cyc.). This is because Ir deposited in the second cycle adheres to the existing single atoms rather than forming new single atoms. As a result, even if the Ir concentration increases, the catalytic activity does not increase unless new single atoms are formed.

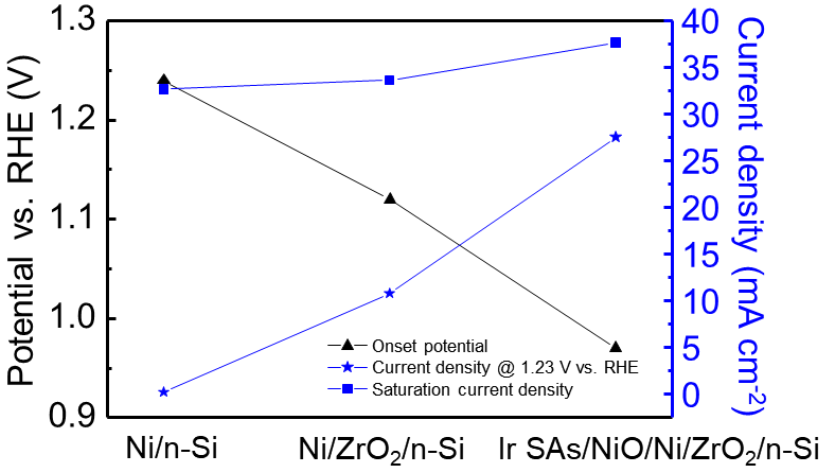

Figure S14. Comparison of the onset potential, current density at 1.23 V vs. RHE, and saturation current density of Ni/n-Si, Ni/ZrO_2/n-Si, and Ir SAs/NiO/Ni/ZrO_2/n-Si.

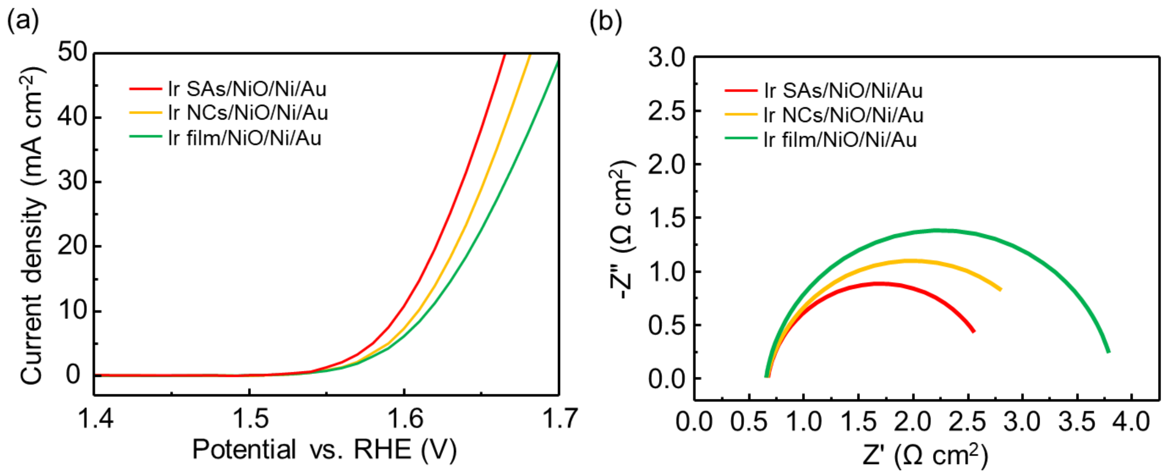

Figure S15. Electrochemical (EC) characterizations of the fabricated anodes with IR-correction. **a** LSV curves and **b** EIS plots of the Ir SAs/NiO/Ni/Au, Ir NCs/NiO/Ni/Au, and Ir film/NiO/Ni/Au anodes.

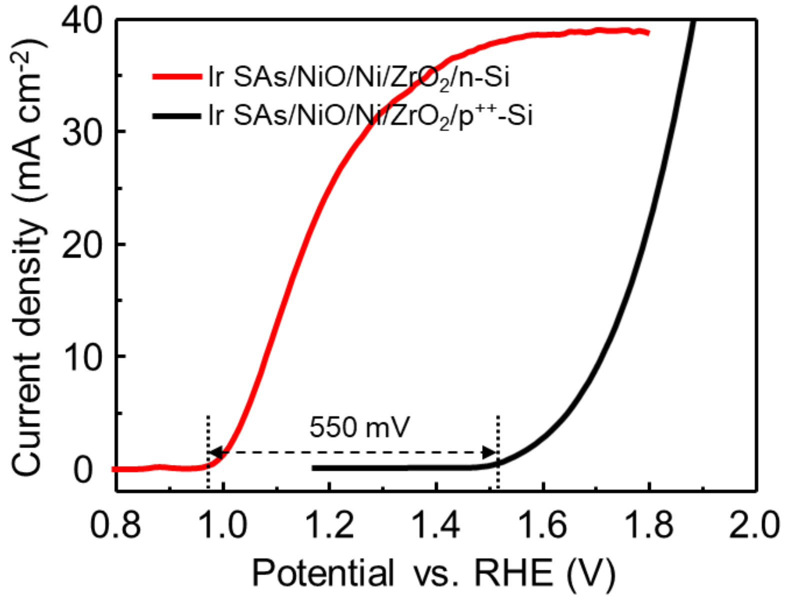

Figure S16. LSV curves of Ir SAs/NiO/Ni/ZrO_2/n-Si photoanode and Ir SAs/NiO/Ni/ZrO_2/p^{++}-Si anode for determining the photovoltage of the device.

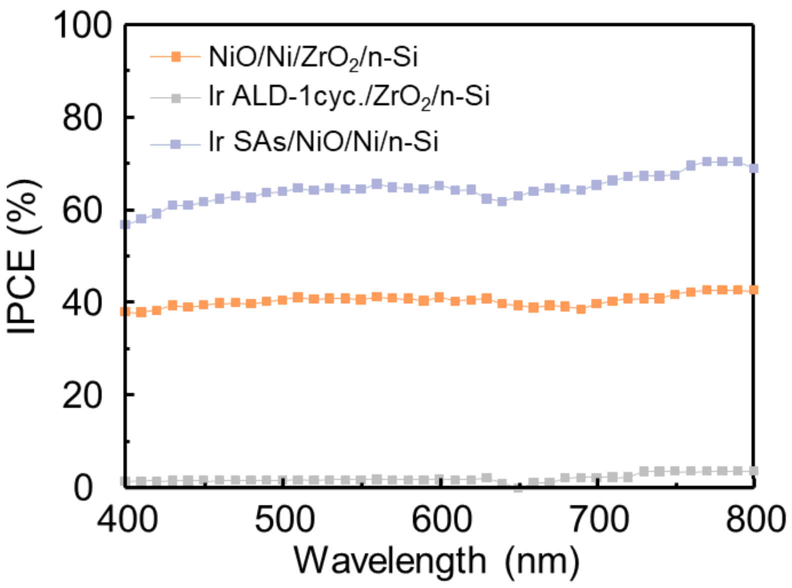

Figure S17. Incident-photon-to-current conversion efficiency of $NiO/Ni/ZrO_2$/n-Si, Ir ALD-1cyc./ZrO_2/n-Si, and Ir SAs/NiO/Ni/n-Si.

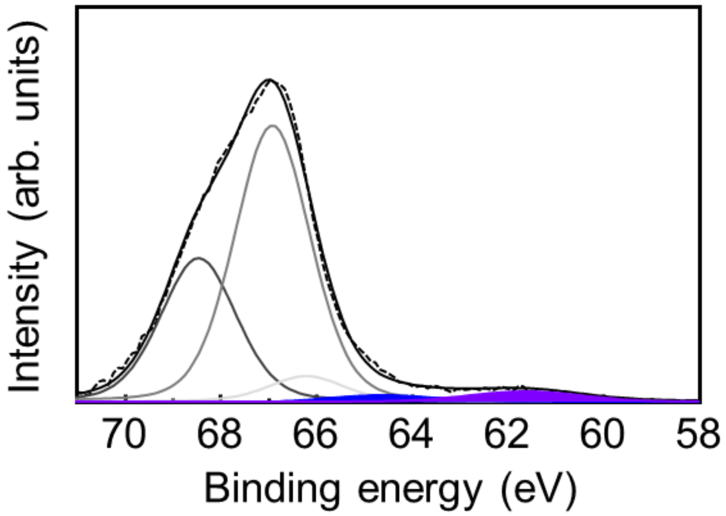

Figure S18. a Ir 4*f* spectra of Ir SAs/NiO/Ni/ZrO_2/n-Si photoanode before performance degradation at 120 h.

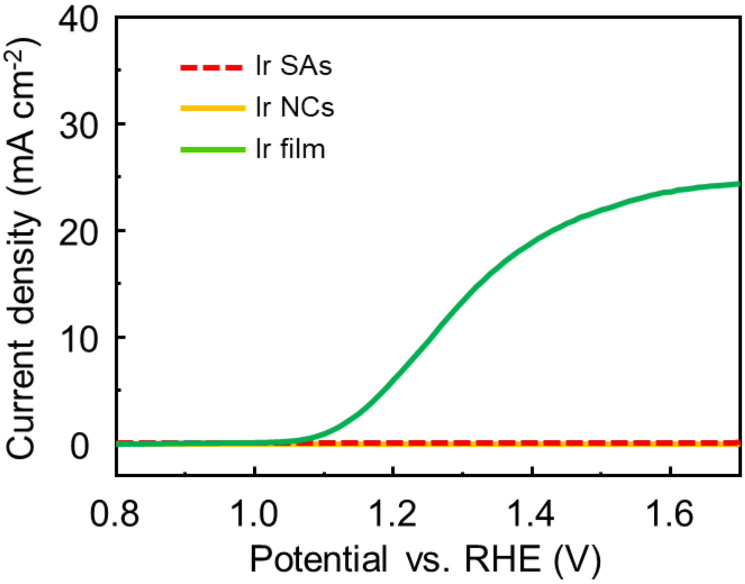

Figure S19. LSV curves of Ir SAs, NCs, and film deposited on $NiO/Ni/ZrO_2$/n-Si photoanodes in acidic condition.

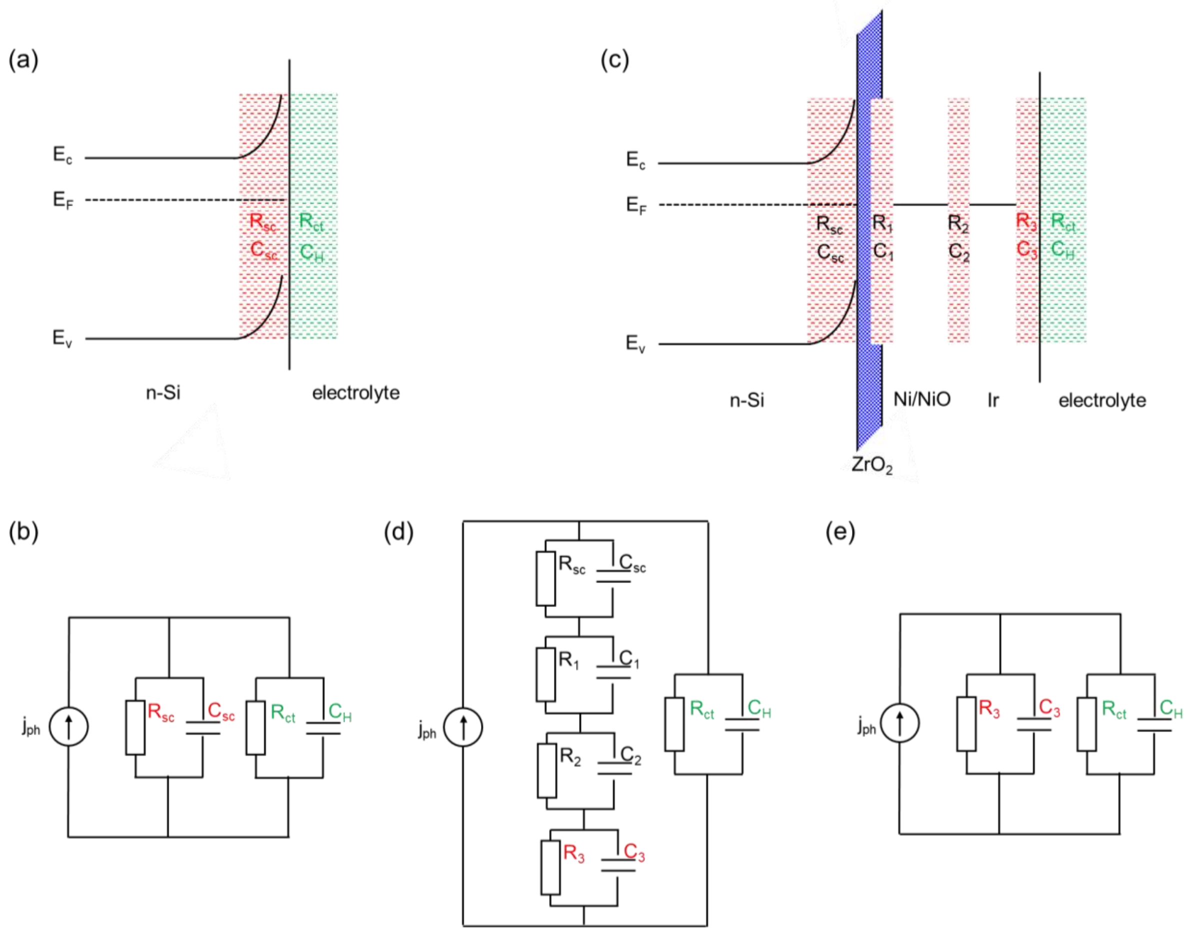

Figure S20. a A generalized theoretical energy band and **b** equivalent circuit model of bare n-Si being in contact with electrolyte for IMPS. **c** The energy band diagram and **d** equivalent circuit model of Ir/NiO/Ni/ZrO_2/n-Si being in contact with electrolyte. **e** The simplified circuit model with the assumption.

A generalized theoretical energy band and equivalent circuit model of bare n-Si junctioned with electrolyte are represented in Supplementary Fig. 18a and b[1]. R_{sc} and C_{sc} are the space charge resistance and space charge capacitance, respectively. These factors are related to surface recombination of photogenerated minority carriers. R_{ct} and C_H are the charge transfer resistance and Helmholtz layer capacitance, respectively. From this model, the values of k_{trans} and k_{rec} were derived as the equation (1) and (2).

$$k_{trans} = \frac{1}{R_{ct}(C_{sc} + C_H)} \qquad (1)$$

$$k_{rec} = \frac{1}{R_{sc}(C_{sc} + C_H)} \qquad (2)$$

For the photoanodes in this study, energy band diagram and equivalent circuit model are provided in Supplementary Fig. 18c and d. There are three additional interfaces compared to

bare n-Si, which are ZrO_2-Ni/NiO, Ni/NiO-Ir, Ir-electrolyte. The resistances and capacitances at each interface is represented by R_1 & C_1, R_2 & C_2, and R_3 & C_3. In terms of surface recombination, not only R_{sc} & C_{sc} but also R_1 & C_1, R_2 & C_2, and R_3 & C_3 contribute to the recombination of photogenerated holes from n-Si. As a result, they can be connected in series as can be seen in Supplementary Fig. 18d.

However, from the EIS data in Figure 4e, we can see that the resistance and capacitance of the interfaces up to the surface are much smaller that those of the interface being in contact with the electrolyte. Moreover, the resistance and capacitance of the interfaces up to the surface are all the same regardless of the samples. Therefore, if we assume that R_{sc} & C_{sc}, R_1 & C_1, and R_2 & C_2 are negligibly small, the circuit is simplified as can be seen in Supplementary Fig. 18e. From this model, the values of k_{trans} and k_{rec} were derived as the equation (3) and (4).

$$k_{trans} = \frac{1}{R_{ct}(C_3 + C_H)} \qquad (3)$$

$$k_{rec} = \frac{1}{R_3(C_3 + C_H)} \qquad (4)$$

Consequently, the charge transfer and charge recombination constants are expressed by the resistances and capacitances at the Ir-electrolyte interface. Therefore, it is possible to compare k_{trans} and k_{rec} depending on the morphology of Ir catalysts regardless of the complicated interface.

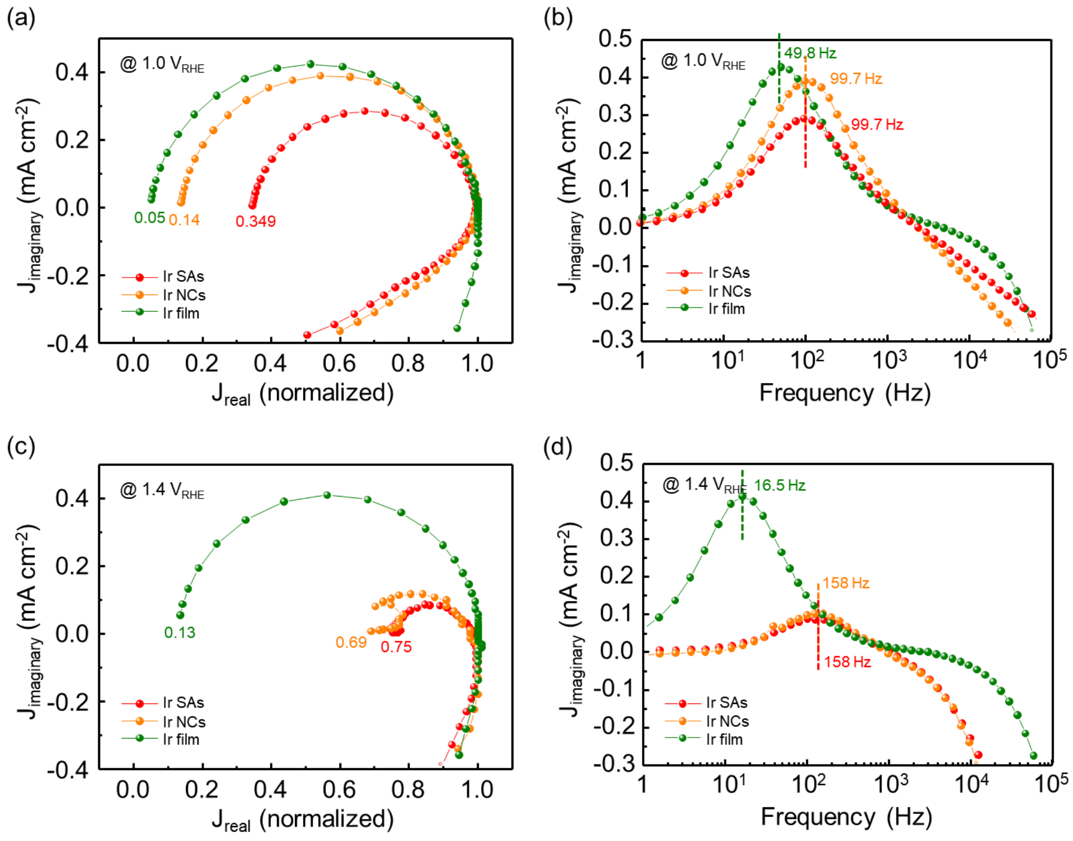

Figure S21. IMPS Nyquist plots showing the imaginary photocurrent vs. the normalized real photocurrent at **a** 1.0 V_{RHE} and **c** 1.4 V_{RHE}. Frequency dependent imaginary photocurrent plots at **b** 1.0 V_{RHE} and **d** 1.4 V_{RHE}.

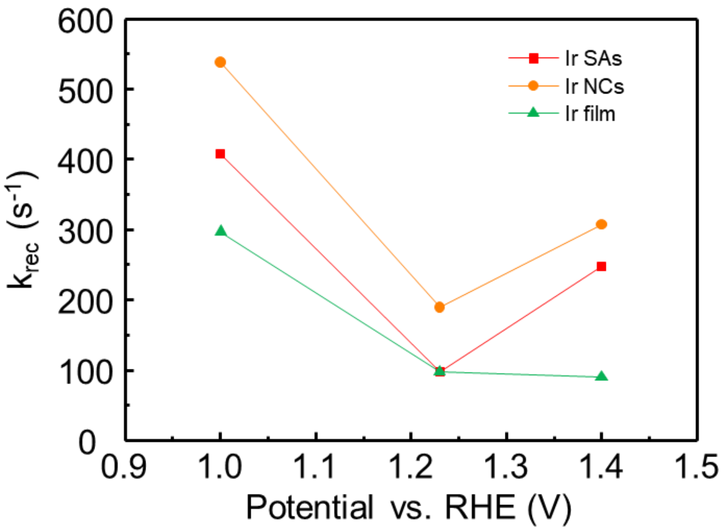

Figure S22. k_{rec} vs. potential vs. RHE (V).

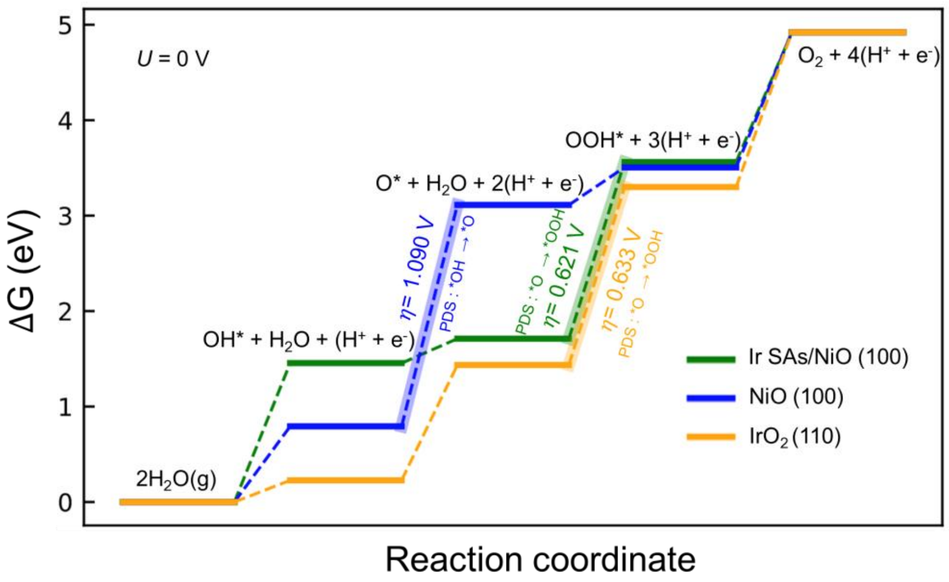

Figure S23. Free energy diagrams of OER at 0 V_{RHE}.

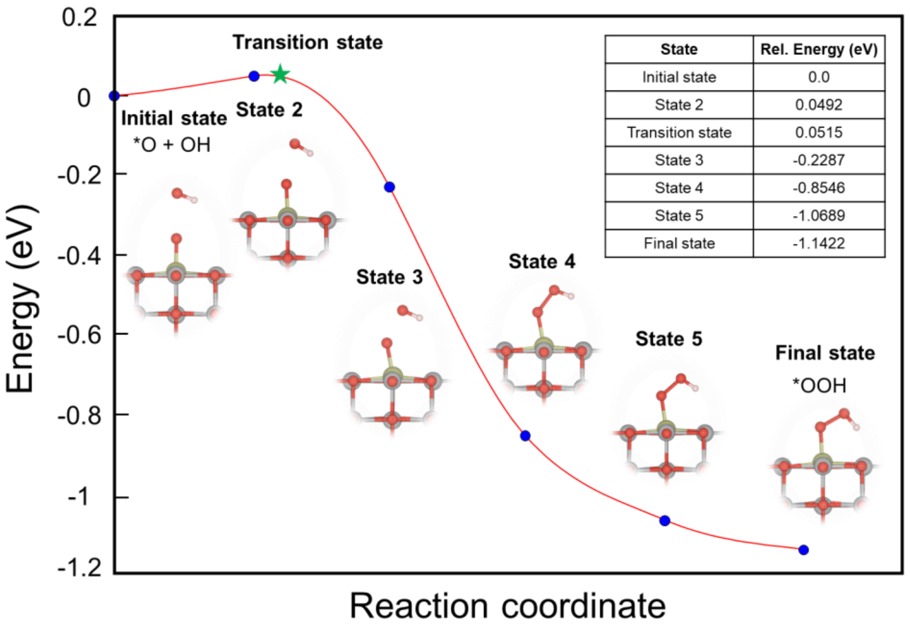

Figure S24. Reaction energy profile of Ir SAs/NiO (100) for *OOH formation calculated with the most favorable incident OH angle.

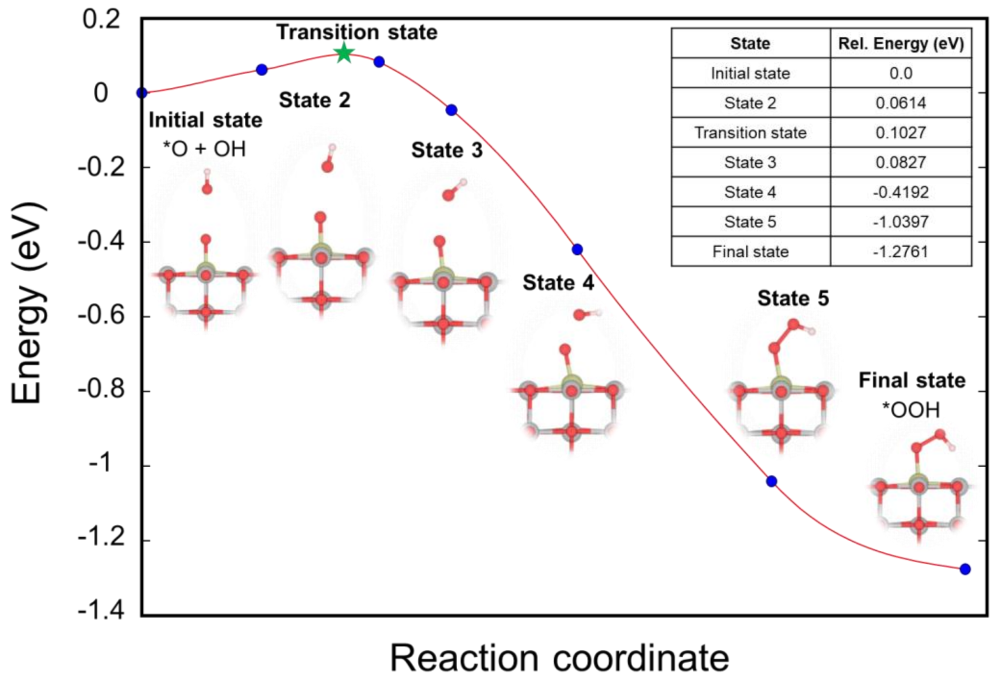

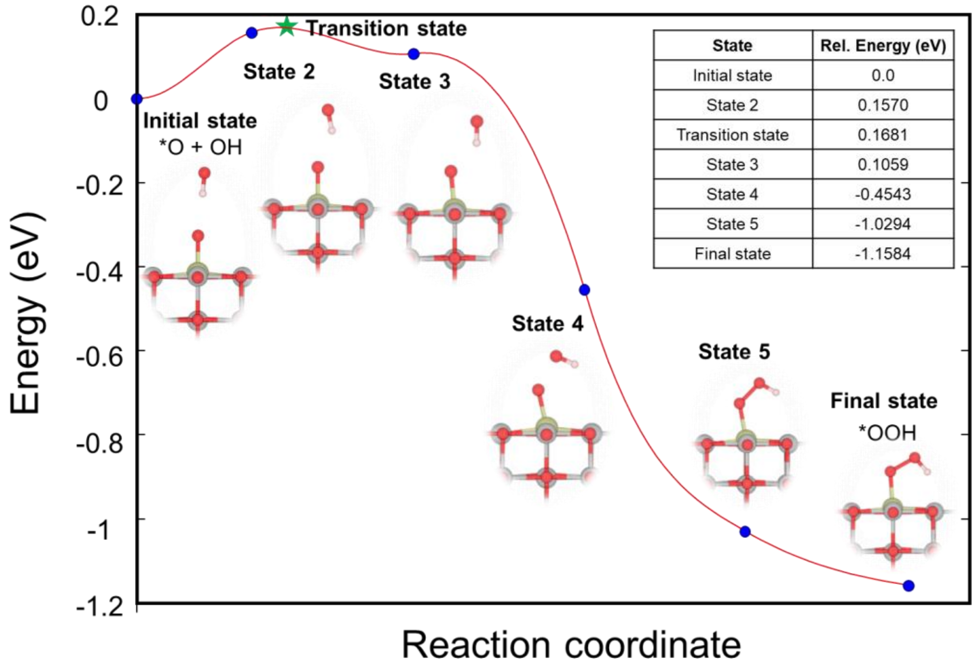

Figure S25. Reaction energy profiles of Ir SAs/NiO (100) for *OOH formation calculated with different incident OH angles.

Table S1. Numerical values of onset potential, saturation current density, and current density at 1.23 V_{RHE}.

Photoanode	Onset potential (V vs. RHE)	Saturation current density (mA cm^{-2})	Current density at 1.23 V_{RHE} (mA cm^{-2})
Ni/n-Si	1.24	33	0.27
Ni/ZrO_2/n-Si	1.12	34	10.82
Ir film/NiO/Ni/ZrO_2/n-Si	1.05	30	11
Ir NCs/NiO/Ni/ZrO_2/n-Si	1	37	22.4
Ir SAs/NiO/Ni/ZrO_2/n-Si	0.97	38	27.7

Table S2. ICP-MS data

Photoanode \ elements	Ir (ppb)	Ni (ppb)	Weight percent (%)
Ir film/NiO/Ni/ZrO_2/n-Si	105.5	156.1	40.33
Ir NCs/NiO/Ni/ZrO_2/n-Si	37.7	166.3	18.48
Ir SAs/NiO/Ni/ZrO_2/n-Si	0.4	93.0	0.43

Table S3. Summary of PEC-OER performance for recently reported Ir-based PEC catalysts applied to Si photoanodes.

Catalyst	Morphology	Si Type	Passivation layer	Electrolyte	J@1.23 V_{RHE} (mA cm^{-2})	Stability (hours)	Reference
**Ir SAs/NiO/Ni**	**SAs + film**	**n-Si**	**ZrO_2**	**1M NaOH**	**27.7**	**130**	**This work**
Ir	film	p^+n-Si	TiO_2	1M NaOH	8	8	Nat. Mater., 2011 [Ref. 2]
$SrCl_2$:IrO_x	film	n-Si	-	0.5M H_2SO_4	20	4	ACS Appl. Mater. I nterfaces, 2020 [Ref. 3]
TiO_2-IrO_x	film	n-Si	-	1M H_2SO_4	7	12	Chem. Mater., 2019 [Ref. 4]
IrO_2-RuO_2	film	n-Si	-	1M H_2SO_4	2	24	ACS Appl. Energy Mater., 2021 [Ref. 5]
IrO_x/Au	film	p^+n-Si	-	1M H_2SO_4	13	0.25	ACS Appl. Energy Mater., 2019 [Ref. 6]
IrO_x/Ir	film	p^+n-Si	-	1M H_2SO_4	12	18	J. Phys. Chem. Lett., 2014 [Ref. 7]

Table S4. Summary of PEC-OER performance for recently reported transition metal-based PEC catalysts applied to Si photoanodes.

Catalyst	Morphology	Si Type	Passivation layer	Electrolyte	J @ 1.23 V_{RHE} (mA cm^{-2})	Stability (hours)	Reference
**Ir SAs/NiO/Ni**	**SAs + film**	**n-Si**	**ZrO_2**	**1M NaOH**	**27.7**	**130**	**This work**
NiCoFe-B_i/NiO/CuO_x	film	n-Si	-	Kb_i+Fe	29	100	Nat. Commun., 2021 [Ref. 8]
Ni	Spiked structure	np^+-Si	SiO_2	1M KOH	20	168	Nat. Commun., 2021 [Ref. 9]
$Co(OH)_2$	film	Black Si	TiO_2	1M NaOH	3	4	Nat. Energy, 2017 [Ref. 10]
Ni	µWs	np^+-Si	-	1M KOH	20	48	Energy Environ. Sci., 2020 [Ref. 11]
				KOH + $[Fe(CN)_6]^{3-}$	20	288	
Ni	film	a-Si/n-c-Si	TiO_2	1M KOH	34	20	Energy Environ. Sci., 2020 [Ref. 12]
NiOOH/Ni	NPs	n-Si	-	1M NaOH	7.8	11	Energy Environ. Sci., 2018 [Ref. 13]
Fe	mPy	n-Si	-	borate buffer (pH=9.6)	0	130	Adv. Energy Mater., 2020 [Ref. 14]
Ni	MWs	n-Si	-	1M KOH	22	8	Adv. Energy Mater., 2019 [Ref. 15]
CoO_x	NWs	n-Si	-	1M NaOH	23.3	20	ACS Catal., 2020 [Ref. 16]
NiO_x/Ni	NPs	n-Si	-	1M NaOH	14.7	2.8	ACS Catal., 2018 [Ref. 17]
NiFe	NPs	n-Si	ZrO_2	1M KOH	26.6	100	ACS Catal., 2018 [Ref. 18]
$Ni_{80}Fe_{20}$	film	n-Si	TiO_2	1M KOH	21.5	20	ACS Catal., 2017 [Ref. 19]
CoVO	film	np^+-Si	-	1M KOH	29.15	3	J. Mater. Chem. A, 2018 [Ref. 20]

NiOOH/NiO_x/Ni	film	n-Si	Al_2O_3	1M KOH	28	80	Small methods, 2019 [Ref. 21]
NiAu	film	n-Si	-	1M KOH	18.8	20	ACS Appl. Mater. I nterfaces, 2018 [Ref. 22]

Table S5. Numerical values of IMPS parameters at an applied potential of 1.0, 1.23, and 1.4 V_{RHE}.

	Photoanode	f at $J_{imag, max}$	Charge transfer efficiency	k_{trans}	k_{rec}
1.0 V_{RHE}	Ir film/NiO/Ni/ZrO_2/n-Si	49.8	0.05	15.64	297.1
	Ir NCs/NiO/Ni/ZrO_2/n-Si	99.7	0.14	87.66	538.46
	Ir SAs/NiO/Ni/ZrO_2/n-Si	99.7	0.35	218.52	407.6
1.23 V_{RHE}	Ir film/NiO/Ni/ZrO_2/n-Si	38.8	0.04	9.75	233.91
	Ir NCs/NiO/Ni/ZrO_2/n-Si	49.8	0.39	123.22	189.52
	Ir SAs/NiO/Ni/ZrO_2/n-Si	38.8	0.6	146.2	97.46
1.4 V_{RHE}	Ir film/NiO/Ni/ZrO_2/n-Si	16.5	0.13	13.47	90.15
	Ir NCs/NiO/Ni/ZrO_2/n-Si	158	0.69	684.65	307.59
	Ir SAs/NiO/Ni/ZrO_2/n-Si	158	0.75	744.18	248.06

Table S6. Exact values of fitted charge transfer resistance.

Resistance / Photoanode	$R_{ct,1}$ [Ω cm^2] (*n*-Si → surface)	$R_{ct,2}$ [Ω cm^2] (surface → electrolyte)
Ni/ZrO_2/n-Si	4.32	73.7
Ir film	10.39	2179.65
Ir NCs	5.53	21.11
Ir SAs	4.36	10.24

Supplementary Notes

Supplementary Note 1. Calculation of k_{trans} and k_{rec}.

<Ir SAs @ 1.23 V_{RHE}>

$k_{trans} + k_{rec} = 2\pi f = 2\pi(38.8) = 243.66$

k_{trans} = transfer efficiency × ($k_{trans} + k_{rec}$) = 0.6 × 243.66 = 146.2

$k_{rec} = 243.66 – 146.2 = 97.46$

<Ir NCs @ 1.23 V_{RHE}>

$k_{trans} + k_{rec} = 2\pi f = 2\pi(49.8) = 312.74$

k_{trans} = transfer efficiency × ($k_{trans} + k_{rec}$) = 0.394 × 312.74 = 123.22

$k_{rec} = 312.74 – 123.22 = 189.52$

<Ir film @ 1.23 V_{RHE} >

$k_{trans} + k_{rec} = 2\pi f = 2\pi(38.8) = 243.66$

k_{trans} = transfer efficiency × ($k_{trans} + k_{rec}$) = 0.04 × 243.66 = 9.75

$k_{rec} = 243.66 – 9.75 = 233.91$

Supplementary References

1. Ponomarev, E. A. & Peter, L. M. A generalized theory of intensity modulated photocurrent spectroscopy (IMPS). *J. Electroanal. Chem.*, **396**, 219-226 (1995).

2. Chen, Y. W. et al. Atomic layer-deposited tunnel oxide stabilizes silicon photoanodes for water oxidation. *Nat. Mater.* **10**, 539-544 (2011).

3. Ben-Naim, M. et al. A spin coating method to deposit iridium-based catalysts onto silicon for water oxidation photoanodes. *ACS Appl. Mater. Interfaces* **12**, 5901-5908 (2020).

4. Hendricks, O. L., Tang-Kong, R., Babadi, A. S., McIntyre, P. C. & Chidsey, C. E. Atomic layer deposited TiO_2–IrO_x alloys enable corrosion resistant water oxidation on silicon at high photovoltage. *Chem. Mater.* **31**, 90-100 (2018).

5. Sahoo, P. P. et al. Si-based metal-insulator-semiconductor structures with RuO_2-(IrO_2) films for photoelectrochemical water oxidation. *ACS Appl. Energy Mater.* **4**, 11162-11172 (2021).

6. Li, L. et al. Operando observation of chemical transformations of iridium oxide during photoelectrochemical water oxidation. *ACS Appl. Energy Mater.* **2**, 1371-1379 (2019).

7. Mei, B. et al. Protection of p^+-n-Si photoanodes by sputter-deposited Ir/IrO_x thin films. *J. Phys. Chem. Lett.* **5**, 1948-1952 (2014).

8. Feng, C. et al. A self-healing catalyst for electrocatalytic and photoelectrochemical oxygen evolution in highly alkaline conditions. *Nat. Commun.* **12**, 1-10 (2021).

9. Lee, S., Ji, L., De Palma, A. C. & Yu, E. T. Scalable, highly stable Si-based metal-insulator semiconductor photoanodes for water oxidation fabricated using thin-film reactions and electrodeposition. *Nat. Commun.* **12**, 1-10 (2021).

10. Yu, Y. et al. Enhanced photoelectrochemical efficiency and stability using a conformal TiO_2 film on a black silicon photoanode. *Nat. Energy* **2**, 1-7 (2017).

11. Fu, H. J. et al. Enhanced stability of silicon for photoelectrochemical water oxidation through self-healing enabled by an alkaline protective electrolyte. *Energy Environ. Sci.* **13**, 4132-4141 (2020).

12. Liu, B. et al. Bifacial passivation of n-silicon metal-insulator-semiconductor photoelectrodes for efficient oxygen and hydrogen evolution reactions. *Energy Environ. Sci.* **13**, 221-228 (2020).

13. Oh, K. et al. Elucidating the performance and unexpected stability of partially coated water-splitting silicon photoanodes. *Energy Environ. Sci.* **11**, 2590-2599 (2018).

14. Oh, K., Dorcet, V., Fabre, B. & Loget, G. Dissociating water at n‐Si photoanodes partially covered with Fe catalysts. *Adv. Energy Mater.* **10**, 1902963 (2020).

15. Tung, C. W. et al. Light-induced activation of adaptive jeunction for efficient solar-driven oxygen evolution: In situ unraveling the interfacial metal-silicon junction. *Adv. Energy Mater.* **9**, 1901308 (2019).

16. Lee, S. A. et al. Amorphous cobalt oxide nanowalls as catalyst and protection layers on n-type silicon for efficient photoelectrochemical water oxidation. *ACS Catal.* **10**, 420-429 (2019).

17. Lee, S. A. et al. Tailored NiO_x/Ni cocatalysts on silicon for highly efficient water splitting photoanodes via pulsed electrodeposition. *ACS Catal.* **8**, 7261-7269 (2018).

18. Cai, Q., Hong, W., Jian, C., Li, J. & Liu, W. Insulator layer engineering toward stable Si photoanode for efficient water oxidation. *ACS Catal.* **8**, 9238-9244 (2018).

19. Cai, Q., Hong, W., Jian, C., Li, J. & Liu, W. Impact of silicon resistivity on the performance of silicon photoanode for efficient water oxidation reaction. *ACS Catal.* **7**, 3277-3283 (2017).

20. Xing, Z. et al. A multifunctional vanadium-doped cobalt oxide layer on silicon photoanodes for efficient and stable photoelectrochemical water oxidation. *J. Mater. Chem. A* **6**, 21167-21177 (2018).

21. Luo, Z. et al. Multifunctional nickel film protected n-type silicon photoanode with high photovoltage for efficient and stable oxygen evolution reaction. *Small Methods* **3**, 1900212 (2019).

22. Hong, W. et al. High-performance silicon photoanode enhanced by gold nanoparticles for efficient water oxidation. *ACS Appl. Mater. Interfaces* **10**, 6262-6268 (2018).
